# Advanced green functional groups for tailoring the membrane features and performance in contaminated wastewater treatment: a comprehensive review

**DOI:** 10.1039/d5ra09447j

**Published:** 2026-05-05

**Authors:** Saba Ahmed Abdulkareem, Khalid T. Rashid, Adnan A. AbdulRazak, Mohammed Ahmed Shehab, Mohammed Nabeel, Mohammed A. Salih, Haidar Hasan Mohammed

**Affiliations:** a Membrane Technology Research Unit, College of Chemical Engineering, University of Technology-Iraq Al Sinaa Street 52 10066 Baghdad Iraq che.24.20@grad.uotechnology.edu.iq Khalid.T.Rashid@uotechnology.edu.iq; b Department of Oil and Gas Refining Engineering, College of Engineering, Al-Turath University Baghdad Iraq adnansss2002@yahoo.com; c Gas Processes and Petrochemicals Engineering Department, Basrah University for Oil and Gas 61004 Basrah Iraq mohammed.ahmed@buog.edu.iq; d Center of Industrial Applications and Materials Technology, Scientific Research Commission Baghdad Iraq mohamedxl2006@gmail.com; e Department of Chemical Engineering and Petroleum Refining, Basrah University for Oil and Gas Basrah 61004 Iraq mohammed.salih@buog.edu.iq; f Thermodynamics and Mathematical Physics Unit, University of Mons 7000 Mons Belgium haidar.alawaad@buog.edu.iq

## Abstract

The need to strike a balance between separation performance and environmental responsibility has led to the development of polymeric membranes with green functional groups as a viable approach for sustainable wastewater treatment. Beyond descriptive reporting, this review critically synthesizes molecular findings connecting membrane structure–property–performance correlations to bio-based functional additives. The effects of naturally occurring functional groups, such as phenolics, flavonoids, polysaccharides, amino-rich biopolymers, and plant-based reactive moieties, on the shape, surface chemistry, and interfacial interactions of the membrane are methodically examined. In order to assess several classes of green additives and fabrication techniques based on their operational stability, durability, sustainability indicators, permeability-selectivity trade-offs, and fouling resistance, a comparative approach is presented. The review clarifies the main mechanisms for antifouling and separation, including hydrogen-bonding networks, surface charge modulation, hydration layer formation, radical scavenging, and antimicrobial activity. It also critically analyzes how these mechanisms result in better dye, heavy metal, and oil contamination removal. Significantly, the analysis reveals the discrepancies and knowledge gaps in published mechanistic interpretations, such as the long-term stability of bio-functionalized membranes under practical operating conditions, the relative contributions of surface chemistry *versus* bulk structural changes, and additive dispersion and leaching effects. Lastly, future research directions are discussed, with a focus on scalable manufacturing routes, hybrid green nanocomposite functionalization, and intelligent and stimulus-responsive bio-additives. All things considered, this review offers a comparative and mechanistic viewpoint that clarifies the actual and potential constraints of green advanced functional groups for developing next-generation high-performance wastewater treatment membranes.

## Abstract 2

Green functional resource for tailoring membrane features and performance in wastewater treatment.

## Graphical abstract

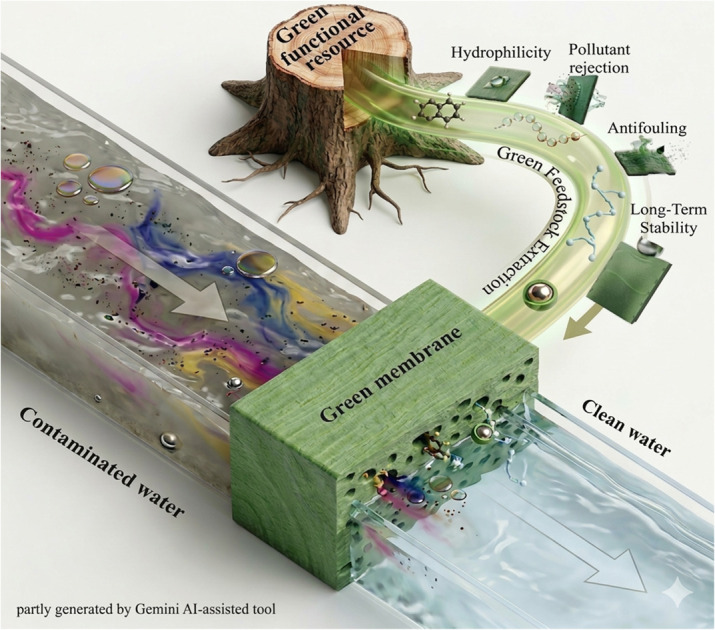

## 1. Introduction

The global water crisis is one of the problems we are currently facing. The increased need for potable water, deteriorating water quality and spread of water-borne diseases pose a serious threat to human existence.^1^ The rapid expansion of contemporary industries and agriculture and the increasing global population have highlighted the fact that numerous countries and regions are confronting the issues of water scarcity and significant water pollution.^2^ Water bodies frequently face contamination as a result of waste discharge from various industrial sectors. This waste may include organic pollutants such as food residues, dyes, pesticides, herbicides, detergents, and pharmaceuticals. Additionally, inorganic pollutants like heavy metal ions and rare earth elements are present, along with other pollutants, including oil spills, grease, and radioactive waste.^3,4^ It is now possible to reuse treated wastewater effluents, and numerous industries employ them in their manufacturing operations.^5^ In order to overcome the difficulties in treating wastewater from printing and dyeing industries, effective pretreatment technologies and innovative treatment technologies that simultaneously manage pollution and effectively achieve the resource utilization potential of these waste streams must be implemented. Pretreatment of printing and dyeing wastewater streams aims to minimize the color, chemical oxygen demand (COD), and biochemical oxygen demand (BOD) of water, remove large particulate matter and modify pH levels. In order to guarantee that discharge regulations are met, advanced treatment techniques are used to further eliminate dissolved contaminants.^6^ Advanced treatment methods are many and varied. They include physical methods (like adsorption and membrane separation),^7,8^ chemical methods (like chemical precipitation and electrochemical oxidation),^9,10^ biological methods (like biofilms and activated sludge processes),^11^ and thermal methods (like crystallization and evaporation).^12^ Each advanced treatment method has its own benefits and drawbacks. A technology for treating wastewater that has developed a lot in the past few years is membrane technology. It has developed rapidly in the last 20 years because it has become very effective at purifying water and wastewater. Owing to its small size, minimal energy demands, and low initial costs, membrane technology has a lot of applications in wastewater treatment.^13^ However, the fouling of membranes limits the effective use of membrane technology. Because fouling reduces the membrane life and consequently degrades membrane performance, it may raise maintenance and operating costs.^14^ The properties of the membrane have a remarkable impact on membrane fouling. Therefore, preparing novel membranes with antifouling characteristics is one of the simplest and most efficient ways to reduce fouling. So far, a number of unique membrane modifications have been developed to change and personalize the surface characteristics and structure of membranes. Typically, membranes are altered to change the hydrophilicity and decrease the roughness of their surface.^15^ Specifically, nanomaterials have been attracting a lot of attention because of their spectacular impact on membrane performance.^16,17^ However, some nanoparticles may be removed from the membrane matrix during the prolonged use of membranes containing nanomaterials. The environmental effects of these nanoparticles have not been fully studied, and some may be harmful. Therefore, it is essential to develop environmentally friendly additives for water treatment membranes.^18^

Before plant-derived membrane additives were widely used, a number of issues needed to be resolved, despite encouraging lab-scale results. By connecting bio-based functional groups to modifications in the membrane structure, surface chemistry, and structure–property performance correlations, this review critically synthesizes antifouling mechanisms. To assess the various classes of plant-based additives and integration techniques in terms of fouling resistance, permeability-selectivity trade-offs, durability, and sustainability indicators, a methodical comparative approach is utilized. The standardization and chemical characterization of plant extracts, discrepancies in the reported antifouling mechanisms, long-term stability and leaching under practical operating conditions, and lifecycle trade-offs compared with traditional inorganic modifiers are among the unresolved issues that receive special attention. This study identifies important knowledge gaps and research goals required for creating scalable, dependable, and truly green membrane technologies by classifying recent publications based on chemical functionality and fabrication methods.

## 2. Membrane separation

Membrane separation technology constitutes up to 53% of the global clean water production processes. It is a cost-effective water treatment method due to its operational simplicity, minimal or no chemical requirements, absence of phase changes, high productivity, scalability, and strong removal efficiency. Because of the previously mentioned traits.^19^ The advancement of membrane technology has considerably benefited desalination and wastewater treatment.^20^ A membrane is described as “a selective physical barrier that, depending on its physical and chemical properties, allows certain compounds to pass through and retains unwanted materials on its surface when a driving force is applied across it”.^21^ Membranes can be divided into two categories: inorganic and polymeric.^22^ Polymeric membranes are becoming more and more popular worldwide^23,24^ because of their increased flexibility, strong film-forming qualities, mechanical strength, chemical stability, high permeation selectivity, and selective chemical species transfer, as well as the low cost of fabrication materials and their necessary pore diameters for different filtration processes.^19^ Researchers have developed various polymeric membranes.^25,26^ Polyethersulfone (PES) and polyvinylidene fluoride (PVDF) are believed to make up the majority of the polymeric membrane market.^27^ Membranes are categorized into four groups based on their pore size and filtration method: microfiltration (MF), ultrafiltration (UF), nanofiltration (NF), and reverse osmosis (RO).^28^ However, because of their inherent hydrophobicity, which favors the adhesion of hydrophobic natural organic materials (NOMs), the development of polymeric membranes is severely hampered by membrane fouling.^23,29^ Therefore, scholars have attempted the hydrophilic modification of membranes *via* several methods.^30,31^

## 3. Challenges, limitations and remedies

**Fig. 1:**
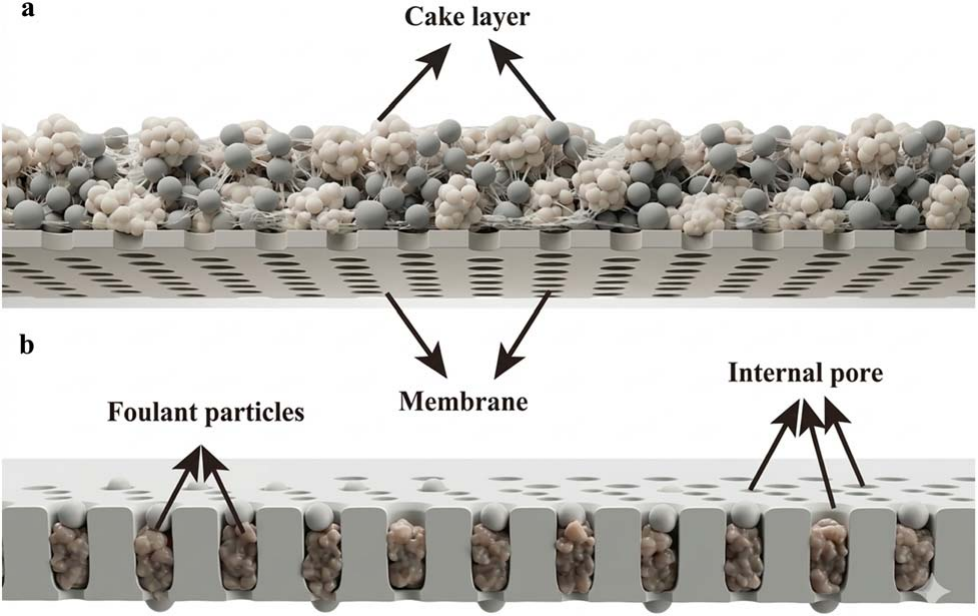
Membrane fouling mechanisms: (a) cake layer formation and (b) pore clogging, partly generated by the Gemini AI-assisted tool.

Membrane fouling is the most difficult challenge in membrane processes for the purification of wastewater and water. Fouling results in a decrease in membrane performance, caused by the deposition of suspended or dissolved solids on the external membrane surface, on the membrane pores, or within the membrane pores. Between filtration stages, the fouling layer is eliminated through cyclic cleaning. Fouling is a severe issue owing to its irreversibility, which means that bacteria or foulants cannot be removed from the membrane surface.^32^ Membrane fouling typically occurs as a consequence of sediment foulant accumulation from wastewater onto the membrane surface, resulting in a “cake layer”, also referred to as external fouling. However, a few small foulant particles penetrate the membrane sublayer and subsequently adhere to the pore walls during the filtration process. Additionally, the foulant materials in the porous support layer can further combine with preabsorbed particulates. This results in internal pollution, which is also referred to as “pore clogging”. Internal fouling is less reversible than external fouling,^33,34^ as illustrated in Fig. 1. The performance of the membrane is significantly impacted by membrane fouling, as it significantly impedes the movement of permeates. Consequently, to ensure the passage of permeates through the membrane, a higher pressure than typical is necessary. The pressure requirement increases as the contamination level increases.^13^ Membrane fouling has significant effects on the overall efficacy of the membrane. These effects include a reduction in the membrane filtration area, an increased downtime, and high energy consumption.^35^ The practical service life of membranes is also reduced by fouling.^20,36,37^ Fouling can be divided into multiple categories, as shown in Table 1, based on the type of foulants.

**Table 1:** Pollution categories in membrane filtration systems

Type of fouling	Characteristics	Ref.
Organic fouling	This form of fouling is second in frequency only to biofouling, and it is characterized by the presence of natural organic matter (NOM) and proteins	38
Inorganic fouling	Also referred to as mineral scaling, it is the accumulation of salts with limited solubility, including CaSO_3_, CaSO_4_, and SiO_2_, on the membrane surface, leading to the formation of rigid scales	39 and 40
Biofouling	It is a result of organisms growing on the membrane surface. Even after pretreatment procedures that can eliminate up to 99.9% of organisms, biofouling continues to be an important challenge in membrane filtration. Microorganisms multiply after adhering to the membrane, which reduces the flow and increases fouling	39
Colloidal fouling	It occurs during the solid removal process when colloidal particles build up on the membrane surface or inside its pores. Colloidal contamination affects the membrane performance primarily by causing particles to aggregate on the surface and form a cake layer. This causes a drop in the power driving the transport of product water, resulting in a decline in flux	41–43

**Fig. 2:**
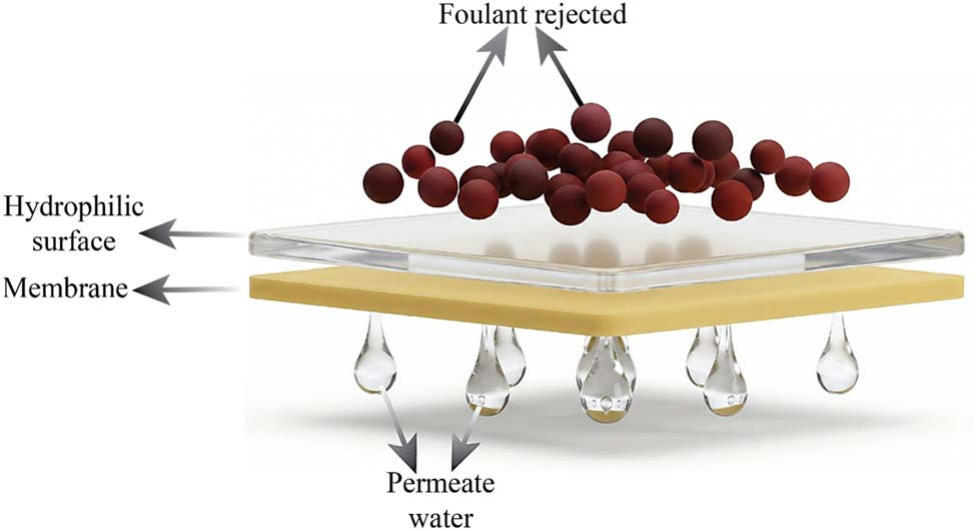
Membrane surface modification, partly generated using the Gemini AI-assisted tool.

Because surface hydrophilicity and roughness significantly influence foulant–membrane interactions, membrane modification is a commonly used technique to reduce fouling. In experiments, hydrophilic and smooth membrane surfaces show less fouling than hydrophobic and rough surfaces (Fig. 2). This is mainly because a stable hydration layer is formed, which inhibits protein and organic adsorption.^44–46^ In modified membranes, recent research has shown stimulus-responsive hydrophilic–hydrophobic switching, where surface wettability is actively controlled by external triggers like pH, ionic strength, and redox conditions rather than happening spontaneously. For instance, zwitterionic functional groups show salt-responsive hydration behavior that has been experimentally connected to decreased protein adsorption and reversible fouling release, whereas catechol- and phenolic-based bio-additives exhibit pH- or oxidation-dependent changes in surface polarity. It has been demonstrated that surface grafting or coating achieved by UV irradiation, plasma treatment, or bio-inspired polymerization introduces functional moieties without changing the bulk membrane structure. On the other hand, bulk modification alters the membrane shape and surface chemistry through radical polymerization or by mixing with hydrophilic additives like green or commercial nanoparticles, hydrophilic monomers, or copolymers.^19,47^ Although redox-responsive switching has been shown to be reversible in controlled laboratory settings, its practical applicability is still unclear because factors like the frequency of redox cycles, the oxidative degradation of functional groups, and long-term stability in real-world water treatment environments are rarely taken into account and need more thorough research.

## 4. Membrane modification strategy

**Fig. 3:**
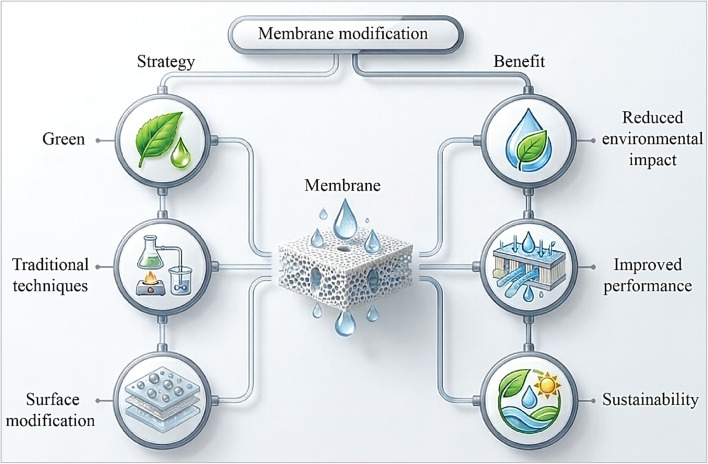
General strategies for membrane modification, partly generated using the Gemini AI-assisted tool.

In order to improve separation performance and sustainability, the general techniques for membrane modification include conventional physicochemical approaches and newly developed green techniques. To modify the membrane structure and surface characteristics, conventional methods such as bulk mixing, surface coating, grafting, and interfacial polymerization have been widely used. Green modification techniques, such as the use of bio-based additives, plant-derived polymers, and naturally occurring nanoparticles and ecologically safe surface functionalization methods, have drawn interest as sustainable substitutes in recent times. The choice of the modification strategy is based on performance goals, material compatibility, fabrication viability, and environmental factors; they are complementary rather than necessary in all situations.^48^ One of the main goals of cutting-edge water and wastewater treatment technologies is to enhance the characteristics and performance of polymer membranes. Improvements usually seek to improve permeability, selectivity, antifouling behavior, and long-term stability while preserving mechanical and chemical resilience. Nanoparticles, green plant-based additives, and functional groups that modify the pore structure and surface chemistry are examples of recent techniques. Under operating conditions, these adjustments increase hydrophilicity, decrease fouling, and encourage higher flow. Furthermore, dynamic interactions with contaminants are made possible by intelligent and sensitive additives, which further enhance the separation efficiency. All things considered, these developments are propelling the creation of more durable, effective, and sustainable membrane systems appropriate for a range of environmental applications, as shown in Fig. 3.^49^

## 5. Green additives

Because of their low toxicity, biodegradability, and natural abundance, green plant-based additives are becoming more and more popular as sustainable substitutes in a variety of industrial applications. These additives, which are made from leaves, roots, oils, and plant extracts, lessen the need for artificial chemicals and their negative effects on the environment. Their use promotes cleaner industrial methods, reduces waste output, and lowers harmful emissions. Plant-based modifiers improve the performance of materials and membrane technology while preserving environmentally favorable characteristics. In general, the application of green plant-based additives promotes safer production methods and supports international initiatives for sustainable industrial development.^49^ Current green methods for the preparation of nanoparticles frequently utilize biodegradable materials, natural substances, harmless solvents, and energy-efficient processes.^50^ Natural materials have special benefits and can be used as additives. For example, the slow-growing, evergreen shrub *Sophora flavescens* is widely utilized in Chinese medicine and has shown antibacterial activity against some barnacles.^51^ Terrestrial plants like green tea also have antibiofouling properties. Kombucha tea, prepared from the leaves of *Malvaviscus arboreus* and *Camellia sinensis*, has demonstrated antibacterial activity against pathogenic fungi due to the high quantities of polyphenols, especially catechins and their derivatives.^52^

**Fig. 4:**
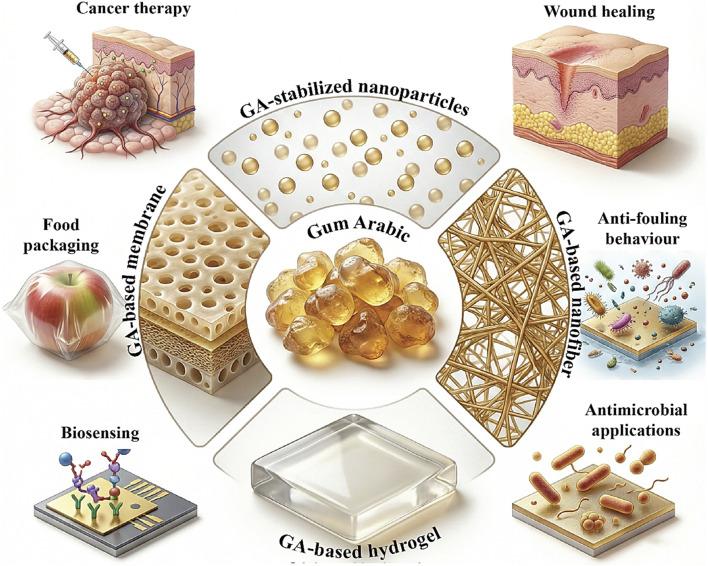
Gum Arabic and its common applications. Adapted from (ref. 56). Copyright © 2025, published by the MDPI. Distributed under the Creative Commons Attribution (CC BY 4.0) license. Modifications were made using the Gemini AI-assisted tool.

By promoting repulsive forces among particles and reducing their propensity to clump together, gum Arabic exhibits superior stabilizing qualities that help to maintain the colloidal stability of NPs.^53^ Additionally, as a nontoxic, biodegradable natural component, the gum Arabic coating acts as a protective barrier that can enhance the biocompatibility of NPs and control their release characteristics.^54^ The wide application range of gum Arabic is shown in Fig. 4. Its distinct physicochemical characteristics allow it to be used in a variety of applications, such as membrane modification, food stabilization, and medicinal formulations. Gum Arabic serves as an emulsifier, a binder, a dispersing agent, and an ecofriendly additive, as seen in the figure. Overall, the wide range of uses shows why gum Arabic is still a useful and versatile natural polymer in contemporary scientific and industrial processes.^55^

**Fig. 5:**
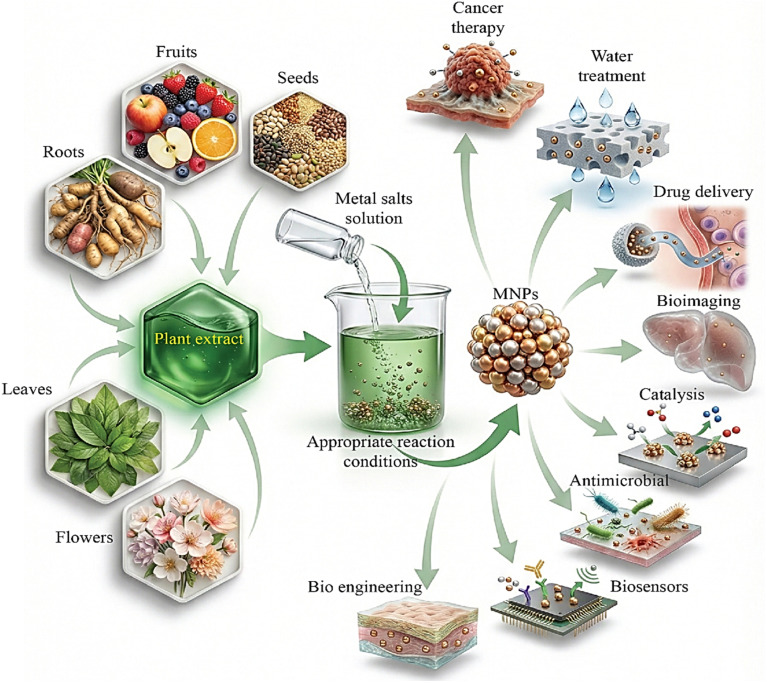
Synthesis of metal nanoparticles from plant extracts and their applications. Adapted from (ref. 60). Copyright © 2021, published by WILEY. Distributed under the Creative Commons Attribution (CC BY 4.0) license. Modifications were made using the Gemini AI-assisted tool.

Green nanotechnology offers tools for converting biological systems to environmentally friendly methods for the synthesis of nanomaterials, offering the best way to lessen the harmful effects of chemical and physical processes and the use of nanomaterials by avoiding any related toxicity, hence reducing the risks associated with nanotechnology. They utilize a variety of resources including plants, algae, and creatures.^57,58^ Plants are preferred over other biosources for the production of metal nanoparticles. Bio-sources play a crucial role in the synthesis of metal nanoparticles, as plant-derived phytoconstituents such as proteins, sugars, terpenoids, alkaloids, and flavonoids act as both reducing and stabilizing agents, enabling efficient bioreduction and enhancing nanoparticle stability.^59^ The use of aqueous solvents is one of the many benefits of plant-mediated synthesis, along with the wide availability of plant materials, their cost-effectiveness, the biocompatibility of plant extracts, and the possibility for large-scale synthesis.^60^ Processes are carried out at room temperature and pressure, saving energy, decreasing hazardous byproduct formation, and avoiding the need for harsh chemicals or extreme conditions.^61^ Because it requires less energy and has a smaller carbon footprint than physical or chemical synthesis methods, the synthesis of nanoparticles from bio-waste is considered a green method.^62^ A variety of methods have been employed by researchers to create nanomaterials from natural plants, including *Hibiscus tiliaceus* extract, *Myrtus communis* L. leaf extract, chamomile flower powder, pomegranate peel extract, grape leaf extract, eucalyptus leaf extract, green tea, *Chenopodium album* extract, and *Moringa oleifera* leaf extract.^63^Fig. 5 illustrates the green synthesis of metal nanoparticles. For example, Roy *et al.* used the fruit extract of *Malus domestica* as a capping agent to synthesize spherical Ag-NPs with an average diameter of 20 nm.^64^ Narath *et al.* also succeeded in using the *Cinnamomum tamala* leaf extract in the synthesis and stabilization of ZnO NPs. This sustainable method provides a better alternative to other conventional methods for ZnO synthesis.^65^ Kumar *et al.* used the leaves of the Andean blackberry (*Rubus glaucus* Benth) to synthesize spherical magnetite nanoparticles (Fe_3_O_4_ NPs).^66^ Furthermore, well-dispersed spherical colloidal gold nanoparticles with average sizes ranging from 17.5 to 23.5 nm were synthesized in water using potato extract (PE) as a reducing and stabilizing agent.^67^ Gold nanoparticles (AuNPs) are characterized by their high stability, controllable size, biocompatibility, and high adsorption capacity, leading to broad applications in drug delivery, biosensors, gene transfer, water purification, cancer treatment, and so on.^59^

## 6. Green plant additives for membrane modification

**Fig. 6:**
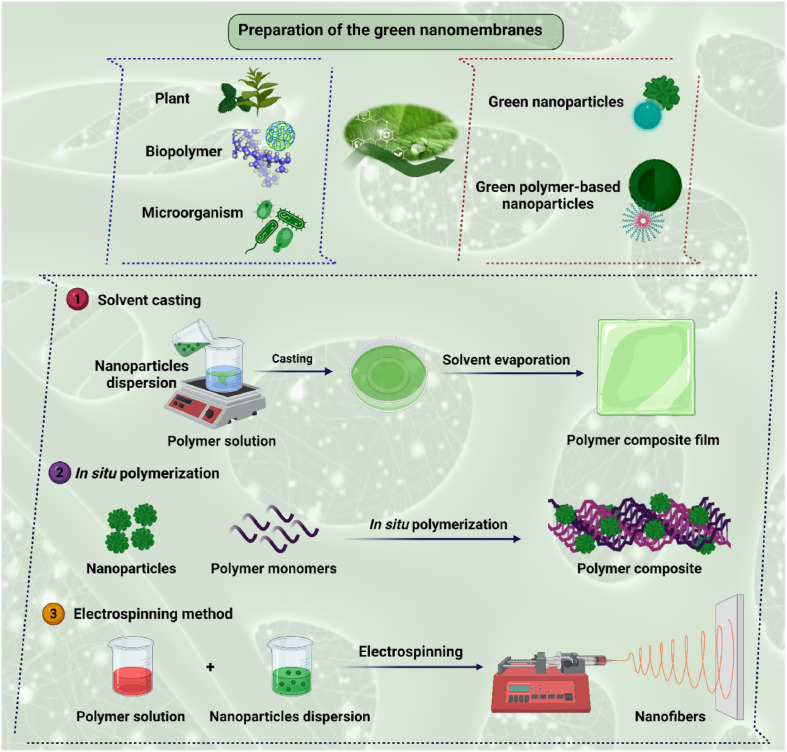
Green membrane modification *via* different green techniques. Adapted with permission from (ref. 70). Copyright © 2023, published by Elsevier.

Green plant additives are environmentally friendly and sustainable modifiers for advanced membrane manufacturing. These additives, which are made from phytochemicals, biopolymers, and natural extracts, lessen the need for hazardous solvents and artificial chemicals. Their addition reduces the environmental impact while improving the membrane hydrophilicity, antifouling behavior, and permeability. Additionally, plant-based modifiers enhance biodegradability and lessen dangerous byproduct formation during membrane manufacturing. Green additives, therefore, present a viable route towards safer, more sustainable, and cleaner membrane technology for water treatment. Several of the antifouling chemicals employed in membrane modification are synthetic compounds that can be harmful to humans and the environment even at trace concentrations. Due to their extensive use and subsequent release as chemical waste during the membrane manufacturing process, these chemicals have a significant toxicological and environmental impact. Consequently, it is important to develop an easy-to-use and ecofriendly replacement technique for modifying membranes in order to increase their fouling resistance without impairing their separation abilities.^68^ Currently, using a natural additive, a green solvent, or a green additive are all possible methods of membrane modification as shown in Fig. 6. Green additives can improve the performance of polymer membranes either in their natural state or after focused modification. When employed directly, plant-based extracts, biopolymers, and natural nanoparticles offer an ecofriendly method to improve hydrophilicity and antifouling behavior. Alternatively, these materials can be chemically or physically refined, such as through functionalization, size reduction, or nanoparticle synthesis, to increase their activity and compatibility. These advances enable more controlled performance benefits, including greater permeability, selectivity, and stability. Overall, both direct and modified green additives promote sustainable engineering of membranes with tunable features.^69^

### 6.1. Processed plant-derived green additives for polymeric membrane modification

**Fig. 7:**
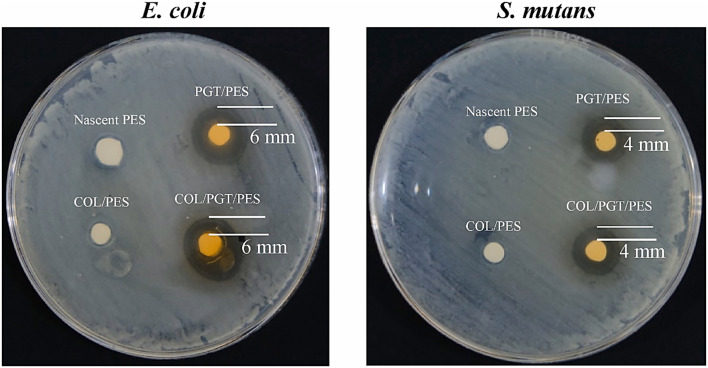
Pure PES, COL/PES, PGT/PES, and COL/PGT/PES membrane antibacterial efficacy against *E. coli* and *S. mutans*. Adapted from (ref. 71). Copyright © 2023, published by Elsevier. Distributed under the Creative Commons Attribution (CC BY 4.0) license.

In order to demonstrate the potential of these natural additives in increasing membrane performance, Ria *et al.* (2023) created PES ultrafiltration membranes incorporated with collagen (COL) and green tea-derived polyphenon-60 (PGT).^71^Fig. 7 shows the membranes' qualitative antibacterial activity according to the inhibition zone approach. Both PGT/PES and COL/PGT/PES membranes show similar inhibition zones against *S. mutans* (≈4 mm) and *E. coli* (≈6 mm). The material achieved over 98% antibacterial efficacy against both bacterial strains, along with approximately 93.5% removal of *E. coli*. The static water contact angle decreased from 65.8° for pristine PES to 37°, indicating enhanced surface hydrophilicity. While dynamic filtration evaluates bacterial elimination during membrane operation, inhibition zones indicate contact-based bacteriostatic effects. These two techniques test the antibacterial efficacy under distinct scenarios. Regarding the polyphenol content, the antibacterial activity of PGT-containing membranes primarily originated from the polyphenolic compounds in green tea, which are known to disrupt bacterial cell membranes. The similar inhibition zones observed for PGT/PES and COL/PGT/PES indicated that the effective polyphenol availability at the membrane surface was comparable and that collagen incorporation did not significantly reduce polyphenol activity. The comparable inhibition zones observed for PGT/PES and COL/PGT/PES indicate that the bioavailable polyphenols at the membrane surface are present at similar effective concentrations, suggesting that collagen incorporation does not adversely affect their antimicrobial activity. Consequently, within the detection limits of the agar diffusion assay, inhibition zone size is not sufficiently sensitive to reflect subtle variations in polyphenol content.^72^

In a recent study, Hansnath Tiwari *et al.* (2023) demonstrated an environmentally friendly method for improving membrane performance by creating polyethersulfone (PES) membranes enriched with silver nanoparticles (Ag NPs) *via* green synthesis using *Mimusops elengi* (bakul) leaf extract. The water permeability of the original PES membrane was 7.61 × 10^−11^ ms^−1^ Pa^−1^, and it decreased to 5.82 × 10^−11^ ms^−1^ Pa^−1^ after adding the Ag NPs. When nanoparticles were deposited on the membrane surface, some pores were blocked, which resulted in a decrease in permeability. Effective antibiotic action against *E. coli* was demonstrated by the resulting membranes.^73^ Noor *et al.* (2021) successfully synthesized Ag-NPs using the aqueous extract of the wild plant *Paronychia argentea* Lam. These green-synthesized nanoparticles were subsequently incorporated into PVDF membranes *via* the phase inversion method to enhance their functional performance. Flux studies showed slight improvements in BSA rejection for PVDF/Ag compared with pure PVDF. However, because of contaminants in the plant extract, the rate of flow of PVDF/Ag was lower than that of pure PVDF. Nevertheless, Ag NPs with a diameter less than 10 nm demonstrated potent antibacterial activity against *E. coli* (G −ve) and *S. aureus* bacteria (G +ve).^74^ Several works that integrate green-synthesized nanoparticles with polymeric membranes are listed in Table 2. These studies show how environmentally friendly nanoparticle synthesis, which frequently makes use of biomass waste or plant extracts, can successfully improve membrane properties. Increased hydrophilicity, mechanical strength, antifouling behavior, and overall filtration efficiency are among the reported improvements. When taken as a whole, these results highlight the expanding potential of green nanotechnology in sustainable membrane engineering.

**Table 2:** Green-synthesized nanoparticles integrated with membranes

Polymer	NP type	Action	Ref.
PVDF	GM-AuNP hydrothermal extract of lambrusco winery grape marc (GM) waste	The modified membrane showed high stability and could be reused for up to 20 cycles without any treatment for 3 months while maintaining the same performance. The photocatalytic performance of the modified membrane was evaluated under natural sunlight irradiation, and complete MB disappearance (100%) was achieved in 116 min	75
PSF	ZnO synthesized using aloe vera (*Aloe barbadensis Miller*)	It exhibited the highest pure water flux (PWF) at 517.9 LMH, with a humic acid rejection of 99.9% and a high porosity of 58.2%. Furthermore, the incorporation of bio-ZnO NPs significantly enhanced antibacterial activity, as evidenced by the increase in the diameter of the inhibition zone from 24.2 to 48.3 mm with increasing bio-ZnO concentrations	76
PES	Bimetallic Fe/Pd synthesized using *Moringa* leaf extract	The water flux increased from 65 LMH to 95 LMH, and dye rejection increased from 86% to 98.4% for NBB and 88.5% to 97.4% for CR compared with neat PES at a dye concentration of 100 ppm	62
PSF	Ag NPs synthesized using *Parkia speciosa* (stink bean) leaf extract	The contact angle decreased from 88° to 65°, and the pure water flux (PWF) increased from 84.2 LMH to 248.7 LMH. The bio-Ag NPs/PSF produced a larger inhibition zone (>6 nm) against *Escherichia coli* (*E. coli*)	77
PSU	Si and Si–Ag synthesized using *Citrus* peels	They achieved higher salt exclusion capacity, as well as better stability over time. They exhibited greater salt adsorption. PSU membranes were evaluated against the ATCC 6633, ATCC 6538, *E. coli*, *Salmonella typhi*, and *Candida albicans* microbes	78
PSF	SiO_2_ from rice husk (RHA)	They resulted in a PWF of 300.50 Liters m^−2^ h^−1^. The highest rejection rate was recorded, reaching 98% for ultraviolet light (UV254) and 96% for dissolved organic carbon (DOC). The modified membranes were recovered at 92% and 96.6%, respectively	79
PVC	TiO_2_ and ZnO synthesized using natural pomegranate plant extract and tangerine plant extract, respectively	The water flux improved to 4.56 L (m^−2^ h^−1^) under solar light irradiation. A humic acid rejection rate of 98.7% was achieved compared with the unmodified membrane	80
PSF	Ag/AgO derived from *Parkia speciosa*	It resulted in increased water flux permeability (393.3 ± 19.7 L m^−2^ h^−1^), humic acid rejection (98.6%), antifouling and the lowest internal resistance (7.9 × 10^12^ m^2^)	81
PES	Silica from rice husk and sugarcane bagasse	It enhanced the WCA angle from 82° to 52°. Also, the silica loading expanded the pore size, resulting in higher permeability	82
PVDF	ZnO NPs derived from the blue–green algae *Arthrospira platensis*	It showed exceptional performance, with a chromium(vi) removal efficiency of 91.69%, an absorption capacity of 10.9 µg cm^−2^, and higher hydrophilicity	83
PES	Ag extract from *Hibiscus rosa-sinensis*	The PWF increased from 11 L m^−2^ h^−1^ bar to 36 L m^−2^ h^−1^ bar, and the WCA decreased from 76° to 61°. Salt rejection also increased, reaching 57% NaCl, 67% MgSO_4_ and 41% CaCl_2_, while the FRR reached 98.7%, compared to 69.09% for pure PES. Ag NPs in PES enhanced dye rejection for MB and Congo red, an anionic dye, likely leading to nearly 100% rejection	84
PVDF	TiO_2_ NPs synthesized using an extract of *Cajanus cajan*	They exhibited higher performance in BSA filtration, increased permeability flux, and a high rejection ratio. The FRR maximum of 96% was achieved, and the contact angle decreased from 85.9° to 69.2°	85

### 6.2. Raw plant-based additives for sustainable membrane engineering

**Fig. 8:**
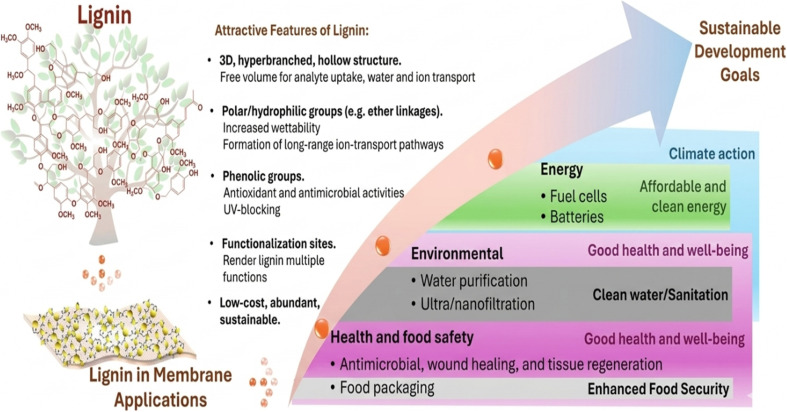
Contribution of lignin to membrane properties. Adapted with permission from (ref. 90). Copyright © 2025, published by Elsevier. Modifications were made using the Gemini AI-assisted tool.

Green bee glue (PRS) nanoparticles from propolis were used to modify polyethersulfone (PES). Propolis was added to the polymer solution in different contents (0, 12.5, 37.5, 62.5, and 87.5 mg). The results showed that 62.5 mg of PRS nanoparticles performed better than others, which resulted in a lower membrane contact angle value of 41.4° compared to pure PES value (64.57°) and a higher clean water flux (∼58 kg m^−2^ h^−1^) and porosity (79%). Furthermore, the PES/62.5 mg PRS showed the maximum rejection rates of 98.8% and 99.8% for both Eriochrome black T (EBT) and Congo red (CR) under a pressure of 1 bar, respectively. These membranes also exhibited high resistance to fouling and self-cleaning capabilities, as demonstrated by a high flux recovery rate (96.83%). When the concentration of PRS NPs increased above 62.5, a decrease in the membrane performance was observed, perhaps because some PRS NPs were scattered irregularly within the PES membrane matrix or some PRS was accumulated on the membrane surface. However, each membrane with PRS NPs outperformed the pure membrane by a significant margin.^86^ Green products like natural polymers show great promise. They are widely available, reasonably priced, and ecofriendly and have a number of uses, including water purification.^87^ One important structural element of plant cell walls is lignin, a significant biopolymer found in nature that offers stiffness, defense, and resistance to deterioration. Lignin, as shown in Fig. 8, is the second most prevalent natural polymer in biomass after cellulose and is essential to preserving the integrity and strength of plants. It is also a great renewable resource for the production of chemicals, innovative materials, and ecofriendly membrane additives due to its rich aromatic composition. Overall, lignin abundance and adaptability demonstrate its significance for sustainable material development as well as natural ecosystems.^88,89^

According to Ming Yong *et al.* (2019), adding lignin to PVC significantly increased membrane wettability and sedimentation resistance, which significantly enhanced overall ultrafiltration performance of PVC. PVC/lignin membranes were manufactured using the reverse phase method. PVC/lignin membranes' morphology and structure were investigated and described. As the lignin content increased from 0 to 50 wt%, the pore diameter and porosity ratio increased from 19.7 to 25.5 nm and from 76% to 84.9%, respectively. The pure PVC membrane exhibited an increase in the contact angle (106.73°) and water flux (PWF; 111.60 L m^−2^ h^−1^) with increasing lignin content. The modified membranes' hydrophilicity was greatly enhanced, their WCA dropped to 41.53°, and their PWF increased to 347 L m^−2^ h^−1^, which is far superior to that of pure PVC. The membrane's ability to remove oils, organic contaminants, and suspended solids also improved. The oil removal rate increased from 68.06% to 97.36%, and the modified membrane showed an FRR of 78% after six cycles of oily wastewater treatment, while the pure PVC membrane showed 49.04%. Therefore, lignin can be utilized to enhance the properties of PVC membranes, and the resulting composite membranes have promising applications in oily wastewater treatment.^91^ Several green additives that have been effectively used to improve the structural and functional characteristics of polymeric membranes are listed in Table 3. These ecofriendly materials enhance properties like permeability, hydrophilicity, and antifouling behavior.

**Table 3:** Growing trend toward incorporating environmentally friendly green materials

Polymer	Additive	Effect on the membrane	Ref.
PLA/PBAT	Banana peel (BP) nanoparticles	They enhanced the PWF and porosity to 105.31 L m^−2^ h^−1^ and 63%, respectively. They exhibited the lowest contact angle (38.99°), the highest oil removal efficiency (96.3%) and a high FRR (93%)	92
PSF	Arabic gum (AG)	Its Congo red (CR) rejection capability was 84.84%, and FRR% was 93.29%. The contact angle was reduced to 59°. It exhibited a high water flux of 8.63 LMH.	93
PES	Dragon blood in resin (DBR)	It exhibited a higher water flux of 246.79 L (m^2^ h^−1^) and a lower contact angle of 52°, along with FRRs greater than 90%	94
PES	Chamomile leaf nanoparticles (Chm NPs)	The water flux (PWF) increased to 498 kg m^−2^ h^−1^, compared with 116 kg m^−2^ h^−1^ for pure PES. The contact angle also decreased from 72° to 47.44°. The rejection of bovine albumin (BSA) exceeded 95%, and the FRR% increased from 52.9% to approximately 93%	95
PES	Acacia gum (AG)	The water flux and porosity of the membrane increased to their highest values by 130% and 77%, respectively. The surface negative charge of the PES/3% AG membrane increased from −11.1 to −24.6 mV	96
PVDF	Ginger extract	The WCA decreased from 92.69° to 84.56°, and the pure water flux increased from 5.07 to 8.82 LMH; also, a significant increase in strength was observed	55
PSF	Lignocellulose from a peanut shell powder as an additive and caramel as a pore-forming agent	The modified PSF showed a significantly increased flux of approximately 250 L m^−2^ h^−1^. Also, BSA rejection was more than 97% with a flux recovery ratio (FRR) of nearly 90%	97
PSF	Rosemary particles	They improved the water flux (from 126 to 359 LMH), decreased the contact angle (from 60.55° to 52.15°) and increased the porosity (from 69.3% to 91.7%). Azithromycin (81.98%), cefixime (58.07%), O II (71.75%), RY 160 (81.71%), and RB 5 (93.69%) were all highly rejected by RE/PSf membranes	98
PAN	Eggplant waste (EGW)	The PWF increased from 136.51 L m^−2^ h^−1^ to 204.71 L m^−2^ h^−1^. Also, greater hydrophilicity, a low contact angle (39.66°), higher porosity (91%), and high tensile strength (8.2 MPa) were achieved. The modified membranes exhibited excellent oil rejection (about 99.95%)	99
PVDF	Tannic acid (TA) as an additive	The PWF increased from 15.4 L m^−2^ h^−1^ to the maximum flux of 50.9 L m^−2^ h^−1^. The FRR of 83% was higher than that of the pure PVDF (58.54%)	100
PSF	Arabic gum (AG)	An oil separation rate of 98% and an FRR of 80% were achieved. Additionally, the mechanical properties improved by 52%. The pure water flux improved to 130 L m^−2^ h^−1^	101
PES	Carrageenan (CAR) derived from edible red seaweed	The contact angle decreased to the lowest value of 48°. A high pure water flux of 1429 L m^−2^ h^−1^ (LMH) was achieved	102
PES	Chitosan (CS) and polyphenon 60 from green tea (PGT)	It achieved high BSA rejection (99.99%), a reasonably high flux recovery (78.78%), and the maximum water flux (46.83 L m^−2^ h^−1^). Additionally, the removal rates of turbidity, aromatic compounds, and chemical oxygen demand (COD) increased from 64.30%, 34.23%, and 62.78% to 88.22%, 64.18%, and 84.21%, respectively. The greatest antibacterial efficacy (99% for *E. coli*) and the largest inhibition zone diameter (7.83 mm for *E. coli*) were attained	103

Sustainability claims in membrane science are typically supported by quantitative and standardized metrics, such as toxicity and additive-leaching assessments, life cycle assessment (LCA) in compliance with ISO 14 040/14 044, and analysis of environmental burdens related to raw material extraction, processing, and end-of-life scenarios. While leaching and ecotoxicity studies are crucial to identify potential environmental concerns emerging from additive release during operation or disposal, the life cycle assessment (LCA) allows a comprehensive evaluation of energy demand, greenhouse gas emissions, and resource consumption throughout the membrane life cycle. Future research should use ecotoxicological analysis to thoroughly confirm the advantages for the environment. Furthermore, bio-additive extraction and functionalization may entail energy-intensive processes and chemical treatments that have an influence on the ecosystem upstream. Sustainability-related claims should be understood qualitatively in the absence of such quantitative evaluations, and thorough LCA and ecotoxicological analyses are advised for future research to thoroughly evaluate environmental advantages.

## 7. Practical challenges and stability considerations

Practical durability and environmental relevance are still understudied in many studies, despite the large body of research on performance improvements attained by the integration of green additives. Oxidative attack, hydrolysis, and mechanical fatigue from fouling and cleaning cycles are known to age and degrade polymer membranes during operation; these processes can change their pore structure, hydrophilicity, and mechanical integrity over time, ultimately affecting flux and selectivity during long-term service. Furthermore, under continuous filtration conditions, additives incorporated in the polymer matrix, particularly inorganic nanoparticles, may leach out; reported instances of nanoparticle loss may have an impact on the effluent quality and membrane performance.^104^ Lastly, most published studies use synthetic dye solutions or model solutes, which do not accurately reflect the complexity of actual wastewater matrices with mixed organic matter, ions, and biological constituents; membranes frequently perform differently when tested under actual effluent conditions, indicating a disconnect between laboratory evaluations and real-world applications.^105^ To increase the translational impact of green additive techniques, it is necessary to perform long-term stability testing, leaching assessments, and validation in actual wastewater systems.

## 8. Comparison of conventional and green additives for membrane performance enhancement

Although conventional membrane materials have long dominated water purification, problems such as fouling, chemical instability, and dependence on dangerous chemicals sometimes restrict their effectiveness. In recent years, green plant-based additives have become popular as sustainable modifiers that can improve the wettability, permeability, and antifouling behavior of membranes. Throughout the membrane life cycle, these bio-derived enhancers lessen environmental toxicity while simultaneously increasing separation efficiency. Comparative analysis reveals a growing transition from traditional membranes to high-performance, environmentally benign filtration systems incorporating green additives (Table 4). This contrast emphasizes how sustainable materials are becoming more and more important for creating the next generation of sophisticated purification membranes.^63,106–108^ Membrane hydrophilicity, permeability, mechanical strength, and antifouling performance have long been enhanced by conventional additives such as inorganic oxides (TiO_2_ and SiO_2_), synthetic polymers, and designed nanofillers. TiO_2_-loaded PVDF composites, for instance, have consistently demonstrated significant improvements in water flux, photocatalytic self-cleaning, and flux recovery following fouling, making them useful for dye removal and oily wastewater treatment applications.^109^ The practical advantages of traditional inorganic fillers for durability and separation performance are demonstrated by the addition of silica nanoparticles to PVC matrices, which also increase porosity and permeability and decrease organic fouling. Nevertheless, these conventional methods may rely on nonrenewable feedstock, potentially dangerous synthesis pathways, and solvents or surface treatments that pose issues for the environment and during scale-up.^110^ To close those gaps in sustainability, green approaches and additives are being developed. Green Ag-NPs have been integrated into polymer membranes to provide their antimicrobial action and fouling resistance while reducing their environmental impact. Plant-mediated or biologically assisted synthesis of metal nanoparticles (*e.g.*, Ag-NPs) produces functional nanomaterials with few toxic reagents and frequently with intrinsic stabilizing biomolecules. Parallel developments improve life-cycle impacts without compromising selectivity or flux using bio-based polymers and more environmentally friendly solvent solutions for membrane casting.^111^ In conclusion, green additives present a viable route towards sustainable, low-impact membrane technologies, even though conventional additives are still useful for enhancing the membrane permeability, selectivity, and fouling resistance. Their adoption promotes environmental safety, lessens dependency on dangerous or nonrenewable chemicals, and may enhance long-term sustainability, a crucial factor as membrane-based water treatment expands internationally.

**Table 4:** Comparison of conventional and green additives for membrane enhancement

Aspect	Traditional additives	Green additives	Representative ref.
Common examples	TiO_2_, SiO_2_, ZnO, CNTs, and synthetic polymers (PVP and PEG)	Plant-synthesized Ag-NPs, ZnO-NPs, lignin, chitosan, gum Arabic, and natural extracts	20, 72 and 88
Synthesis route	Chemical/thermal processes using surfactants and catalysts	Plant-mediated or biological synthesis using extracts and low-toxicity routes	112 and 113
Environmental impact	May generate hazardous solvent waste and nonrenewable inputs	Ecofriendly, renewable, and biodegradable byproducts	101 and 105
Effect on hydrophilicity	Strong improvement *via* metal oxides and hydrophilic fillers	Enhanced by natural functional groups (phenolics, flavonoids, and polysaccharides)	19 and 58
Antifouling performance	Excellent photocatalytic fouling reduction (*e.g.*, TiO_2_)	Strong antibacterial/antifouling effects from plant compounds	72 and 114
Mechanical properties	High mechanical reinforcement and improved tensile strength	Moderate improvement; depends on the biomass composition	20 and 78
Cost and availability	Higher cost for engineered nanoparticles	Low cost and abundant renewable materials	78
Long-term risks	Possible nanoparticle leaching and environmental persistence	Lower toxicity and reduced leaching due to organic stabilizers	115 and 116
Scalability	Industrially established	Promising but requires synthesis standardization	16, 17 and 101
Typical performance outcomes	High flux, strong antifouling, and durable	High hydrophilicity, antibacterial activity, and environmentally safe	19, 72 and 88

## 9. Smart and responsive green additives

The next development in sustainable membrane engineering is represented by smart and responsive green additives, which combine the usefulness of stimulus-responsive systems with the environmental advantages of bio-derived materials. Smart green additives can actively react to changes in the pH, temperature, light, or ionic strength, in contrast to conventional plant-based or natural additives, which passively improve hydrophilicity or antifouling qualities. Membranes may adjust in real time to changing feedwater conditions thanks to this dynamic behavior, which improves selectivity, lowers fouling, and increases long-term operating stability. When functionalized or coupled with bio-inspired motifs, biopolymers, including chitosan, alginate, tannic acid, and cellulose derivatives, provide reversible swelling, charge-switching, or conformation modifications that control water transport and pollutant interactions. Incorporating these clever green materials into polymeric membranes offers a new route towards high-performance, energy-efficient, and self-regulating separation technologies, in addition to being consistent with circular and ecofriendly design concepts. As research progresses, smart green additives are anticipated to be a key component in the creation of next-generation membranes that are suited to intricate and changing water treatment problems.^117^

## 10. Mechanism

Through processes that activate in response to certain environmental stimuli, smart and responsive green additives improve membrane performance and allow membranes to dynamically adapt during filtration. The chemical functionality of bio-based compounds, including polysaccharides, polyphenols, and bio-inspired moieties, as well as their capacity to experience reversible structural or charge changes, gives rise to these mechanisms. The following are the important mechanisms:^118–120^

### 10.1. Functional group ionization that responds to the pH

Functional groups (–COOH, –OH, –NH_2_, and phenolic groups) found in many green additives undergo protonation or deprotonation based on pH. These groups become protonated at low pH, which lessens the negative charge and electrostatic repulsion between foulants and the membrane surface. Deprotonation enhances hydrophilicity and repels charged impurities by increasing the surface charge density at high pH. The membrane can transition between compact and swollen states thanks to this reversible ionization, which enhances pollutant rejection and permits natural self-cleaning.

### 10.2. Changes in the temperature-responsive conformation

Certain bio-based additives show thermo-responsive behavior, in which temperature changes cause polymer chains to expand or contract. Hydrophilic groups dominate below a certain temperature, improving permeability and water uptake. Hydrophobic interactions intensify above this temperature, tightening the pore architecture and decreasing fouling. Membranes can control the flux based on the temperature thanks to this process.

### 10.3. Structural rearrangement that responds to redox

Green additives that are rich in phenolic compounds, such as catechols and tannic acid, can undergo reversible oxidation and reduction, allowing surface charge, crosslinking, or switchable adhesion. Quinone formation *via* oxidation encourages surface stiffness and crosslinking. Reduction increases hydrophilicity and protein-repelling behavior by restoring catechol groups.

### 10.4. Reversible switching between hydrophilicity and hydrophobicity

In reaction to environmental stimuli, certain smart additives change the surface energy of the membrane. Increased hydrophilicity during filtration (better flow) and increased hydrophobicity after filtration (better fouling release) are the two possible outcomes.

## 11. Thermo-responsive behavior in green membranes

Synthetic polymers with a sharp, lower or upper critical solution temperature (LCST/UCST), like poly(*N*-isopropylacrylamide) (PNIPAM), are traditionally linked to thermo-responsive behavior in membrane systems, where reversible hydration–dehydration transitions result in noticeable changes in wettability and permeability. Conversely, the majority of native biopolymers (*e.g.*, cellulose, chitosan, alginate, starch, and proteins) lack sharp and reversible LCST/UCST transitions in water due to their rigid hydrogen-bonded structures, wide molecular weight variability, and strong intermolecular forces.^121,122^ Therefore, thermo-responsiveness in so-called green or bio-based membranes usually results from bio-synthetic hybrid systems, in which thermo-responsive synthetic segments (most often PNIPAM or PNIPAM-like moieties) are chemically grafted, blended, or copolymerized with biopolymers acting as sustainable backbones or supports. The temperature-triggered switching capability of these systems is provided by the grafted synthetic component, while the biopolymer provides mechanical stability, hydrophilicity, and environmental compatibility. Therefore, reported thermo-responsive effects in green membranes are engineered properties rather than inherent characteristics of unmodified biopolymers, and their practical applicability depends on the grafting density, cycling stability, transition sharpness, and long-term resistance to oxidative and thermal degradation.^123,124^

## 12. Functional group chemistry and its role in membrane performance

The functional group chemistry introduced in the membrane matrix can be used to explain the observed antifouling and separation performance. The observed antifouling behavior and separation efficiency can be fundamentally attributed to the tailored functional group chemistry incorporated within the membrane matrix. Strong hydrogen bonds with water molecules are encouraged by hydrophilic moieties like hydroxyl and carboxyl groups, which result in the creation of a stable hydration layer at the membrane surface. This hydration layer improves antifouling behavior by acting as an energy barrier against microbial adhesion and the adsorption of organic foulants. Molecular level interactions orchestrate the balance between permeability and selectivity by modulating surface charge *via* ionizable functional groups, which repel similarly charged foulants, minimize pore blockage, and enhance contaminant rejection. Concurrently, intrinsic antibacterial and radical-scavenging properties suppress oxidative degradation and biofilm formation, preserving pore integrity and sustaining long-term membrane performance.^125,126^

## 13. Outlook, challenges and future research direction

Environmentally benign membrane modification techniques have drawn a lot of interest as efforts to replace dangerous membrane modifiers with safer and more sustainable alternatives intensify. Before such membranes can be extensively used, a number of significant obstacles must be overcome, despite encouraging developments utilizing bio-based additives, green solvents, and environmentally friendly surface treatments. It is particularly challenging to quantitatively validate sustainability claims regarding energy usage, emissions, toxicity, and end-of-life impacts because there are currently few comparative life cycle assessment (LCA) studies on green membranes than on conventional membranes. Furthermore, there are still obvious trade-offs between performance and sustainability because improvements in hydrophilicity and antifouling behavior may jeopardize chemical stability, mechanical strength, or long-term durability under practical operating conditions. The majority of antifouling chemicals used today are synthetic and perhaps dangerous, which raises issues for wastewater contamination, occupational exposure, and the release of nanoparticles during production, use, and disposal. On the other hand, natural or green additives like plant extracts, biopolymers, and materials produced from waste offer low-toxicity, renewable substitutes that frequently have several uses, such as metal-binding ability, hydrophilicity, antibacterial activity, and antioxidant behavior. Nevertheless, insufficient mechanistic knowledge of long-term fouling resistance, membrane aging, biodegradation, and additive leaching prevents their practical implementation. Membrane integrity can be compromised, permeability reduced, and performance unpredictable due to nonuniform dispersion within polymer matrices, heat and solvent instability during phase inversion, and agglomeration at high loading levels. Large-scale extraction and purification may be time-consuming and costly, and variability in natural feedstock brought about by source, seasonality, and extraction techniques creates repeatability issues. From a techno-economic standpoint, systematic cost analysis, supply chain evaluations, and scalability assessments are still hard to come by. Therefore, controlled additive integration techniques, hybrid systems that combine green materials with small amounts of safe synthetic modifiers, and standardized LCA and eco efficiency frameworks should be the main areas of future research. End-of-life factors, such as recyclability, biodegradability, and safe disposal routes, are just as crucial as application-specific validation with actual wastewater streams or industrial effluents. In order to convert laboratory-scale green membranes into high-performing, financially feasible, and really sustainable technology appropriate for practical environmental applications, these gaps must be filled.

## 14. Conclusion

This paper discusses the use of membranes in water treatment, reviewing the various methods used to modify them to improve permeability and reduce surface fouling. These methods include traditional approaches such as blending, surface coating, and grafting, which have contributed to improving the operational properties of membranes. However, due to the drawbacks of traditional methods, researchers are focusing on biological systems and preferring green synthesis. Recent studies have focused on the use of environmentally friendly materials as additives in membrane development due to their biodegradability, low toxicity, and natural availability. Some of these materials include plant extracts, biopolymers, and green nanoparticles, which can improve hydrophilicity and antifouling capabilities without causing environmental damage. This represents an important step toward designing sustainable membranes that combine high efficiency with environmental friendliness, making it one of the most promising areas in water treatment. However, studies on this topic are still limited and have not addressed all aspects related to the working mechanism and the relationship between the chemical composition of plants and the final properties of membranes. Because of the wide range of green materials, further in-depth studies are still needed to better understand their potential and expand their application in developing membranes for water treatment technologies.

## Conflicts of interest

The authors declare no conflicts of interest.

## Data Availability

No primary research results, software or code have been included, and no new data were generated or analyzed as part of this review.
